# Supplementary material for: Developing political-ecological theory: The need for many-task computing
Source: PLoS One. 2020 Nov 24;15(11):e0226861. doi: 10.1371/journal.pone.0226861 (PMC7685461; doi:10.1371/journal.pone.0226861)
Supplement: S1 Appendix — Shell scripts to initiate a Gigaspace, guidance for running on a shared cluster computer, and documentation of the cheetah EMT simulator. (PDF) [file pone.0226861.s001.pdf]

Appendix A: Scripts to launch a JavaSpaces job

At a command prompt on a cluster computer running Unix/Linux, type the command

`sh pesrun.shl`

to run the shell script `pesrun.shl` (Figure 1). This script submits the script `pesmaster.shl` to the batch job scheduler (Figure 3) which in-turn

1. starts a GigaSpace by running the script `gspace.shl` (Figure 2),
2. starts the workers by submitting several instances of the script `pesclient.shl` to the scheduler (Figure 4), and
3. starts the master with the JAVA™ command at the end of the script.

```
qsub -d /home/haas/sanparks -q hotel -m e -l nodes=1:ppn=3 \
   -l walltime=10:00:0 -o shell.out -e shell.err pesmaster.shl
```

Fig 1. The script `pesrun.shl` that is used to submit a JavaSpaces job.

```
rh="/projects/builder-group/jpg/gigaspaces/bin"
myconfigs="/home/haas/jsutils"
host=$(hostname)
cd /home/haas/jsutils
#
# Start a GigaSpace.
#
sh ${rh}/space-instance.sh -name space671 &
echo - GigaSpace Running -
```

Fig 2. The script `gspace.shl` that is used to start a GigaSpace (a form of a JavaSpace).

```
#!/bin/sh
#
# The name, "pesmaster.shl" stands for
#       "political-ecological simulator -- master"
#
# This script runs a JavaSpaces program as one master node and several
# client (worker) nodes.
#
# First, start a GigaSpace
#
sh /home/haas/jsutils/gspace.shl
sleep 5
#
#Get this node's IP address.
#
mastername=$(hostname)
echo Master is: $mastername
#
# Start "nmclients" clients.
# Make sure ppn is set to the number of threads in Id.java.
#
nmclients=2
for ((i=1; i <= nmclients; i++))
do
   outfile="worker"$i".out"
   errfile="worker"$i".err"
   thisjob=\$(qsub -d /home/haas/polbio -q hotel -m n \
            -l nodes=1:ppn=3 -l walltime=1:00:0 -o $outfile -e $errfile \
            -v ARG1=$i -v ARG2=$mastername \
            pesclient.shl)
done
#
#Submit master job.
#
ar="/projects/builder-group/jpg/gigaspaces"
arj="/projects/builder-group/jpg/java"
# Note that "*" is to match .jar files only in the -cp option.
/usr/lib/jvm/java/bin/java -Djava.rmi.server.useCodebaseOnly=false
   -Djava.security.policy=/home/haas/id/policy.all
   -Djava.rmi.server.RMIClassLoaderSpi=
   net.jini.loader.pref.PreferredClassProvider
   -cp "/home/haas/id:${arj}/*:${ar}/lib/optional/*:${ar}/lib/platform/*:
      ${ar}/lib/required/*:${arj}/derby/lib/derby.jar"
   -Xms12024m -Xmx12024m Run\_id eastaf.id master $mastername
   --max-mem-size=12024m
```

Fig 3. The script `pesmaster.shl` that is used to spawn clients (workers) and start the master. The file, `eastaf.id` is the file that software package, **id** reads in order to execute the requested analysis, see Appendix B.

```
#
# Starts a client version of id.
#
ar="/projects/builder-group/jpg/gigaspaces"
arj="/projects/builder-group/jpg/java"
/usr/lib/jvm/java/bin/java -Djava.rmi.server.useCodebaseOnly=false
   -Djava.security.policy=/home/haas/id/policy.all
   -Djava.rmi.server.RMIClassLoaderSpi=
   net.jini.loader.pref.PreferredClassProvider
   -cp "/home/haas/id:${arj}/*:${ar}/lib/optional/*:${ar}/lib/platform/*:
      ${ar}/lib/required/*:${arj}/derby/lib/derby.jar"
   -Xms12024m -Xmx12024m Run\_id eastaf.id client $ARG2 --max-mem-size=12024m
```

Fig 4. The script `pesclient.shl` used to start a client.

Appendix B: Running a JavaSpaces program on a cluster computer

Many cluster computers are *non-dedicated* (see for example, [1]) i.e., computing resources are shared by the users. The cluster computer's *job scheduler* e.g. the TORQUE Resource Manager [2] employs algorithms to assign user jobs to either compute nodes or to queues. These algorithms assign jobs by balancing (a) the need to make efficient use of the computer, (b) the need to give each job the resources (processors and memory) it requires, and (c) the need to enforce "fairness" policies. For example, an individual user's limit on the Triton Shared Computing Cluster (TSCC) at the San Diego Supercomputer Center (SDSC) is 128 cores simultaneously across all allocated compute nodes (there, each compute node contains 16 cores).

Scheduling a compute job that utilizes the JavaSpaces technology on a cluster computer involves a request for one master and n_W worker compute nodes. See Appendix A for the four scripts that can be used to submit a JavaSpaces job to a cluster computer. If there were no other users, one could run 128/9=14 cores per compute node in an eight-worker JavaSpaces program. But such a job would wait in the computer's queue a long time because, under the scheduler's "fairness" policy (see for example, [3]), such a job would be given low priority. This is because the PBS "fairness" rule attempts to level the amount of compute time each user enjoys over some time period, e.g. one hour. One way to avoid such latency is to limit the number of requested cores per compute node to no more than about three. Monte Carlo simulation is used by **id** to approximate solutions to bayesian belief networks. Because each Monte Carlo realization of a bayesian belief network is independent of the others, the total number of requested realizations is evenly divided across the requested number of cores on a compute node. This means that the simulation of a bayesian belief network will run faster if more cores per compute node are requested. Consequently, there is a tradeoff between queue wait time and objective function evaluation speed.

To manage compute cycle expense, the maximum number of objective function evaluations can be specified in the **id** command file. Once this limit is reached, **id** terminates the MDAS algorithm. For example, say that it is desired to restrict a simulator job to no more than five hours of wall clock time. If an objective function can be evaluated in one minute, then an eight-worker JavaSpaces MTC application should be restricted to terminate the MDAS algorithm after $60 \times 5 \times 8$ function evaluations (number of function evaluations in one hour by one worker $\times$ number of hours that worker is allowed to work $\times$ number of workers).

Appendix C: Documentation for the cheetah EMT simulator

This documentation follows the Dahlem protocol given in [4] for documenting an agent-based model.

Overview

Rationale

Conservation policies need to be based on reliable predictions of the effect that different policies might have on a managed ecosystem. Models can provide such predictions. One way to model such a political-ecological system is to first, build submodels of all ecosystem-impacting groups and a submodel of the managed ecosystem, and then, have these submodels interact with each other through time. A proposed policy is expressed by applying its opportunities and constraints to the group submodels. Finally, the effectiveness of this policy is evaluated by observing how the ecosystem submodel responds to the sequence of actions issued by the policy-manipulated groups.

Agents

This simulator focuses on the sustainable management of the cheetah meta-population across the East African countries of Kenya, Tanzania, and Uganda. Each of these countries contains four group submodels: the presidential office, the environmental/wildlife protection agency, its rural residents, and its pastoralists, respectively. The last group submodel captures the actions of all conservation NGOs that have operations in at least one of these countries.

Other entities

A system of stochastic differential equations (SDEs) is used to model the managed ecosystem. Here, this is the ecosystem that hosts cheetah and their prey within the countries of Kenya, Tanzania, and Uganda.

Boundaries

The spatial boundary is the contiguous region enclosed by the country boundaries of Kenya, Tanzania, and Uganda. The temporal boundary is from the year 2002 to the year 2030.

Relations

Cheetah abundance is affected by poaching, and by loss or gain of habitat. Antipoaching units have law enforcement authority over poachers. Poachers directly interact with the ecosystem.

Activities

Poachers either poach cheetah and/or their prey or refrain from doing so. Antipoaching units either shoot armed poachers, arrest poachers, or refrain from doing either. Cheetah and prey habitat is affected by decisions made by a country's presidential office to either *degazette* land that had been a wildlife reserve, or *gazette* new land for a wildlife reserve.

Design Concepts

Time, activity patterns and activation schemes

The political-ecological system simulator is based on a bulletin board (also referred to as a *blackboard*) message posting architecture. A typical simulation run is over three years with a time step of 12 weeks. Each group submodel reacts to an action directed towards it by another group by posting on the bulletin board a decision option selected from a pre-specified, finite list of options.

Interaction protocols and information flows

The simulator operates as follows. First, a seed *action message* is posted to the bulletin board. This message consists of the time, the actor's name, the target's name, and the *ecosystem management action taxonomy* (EMAT) action code [5, ch. 8]. Next, each group reads this message and, after determining their preferred action-target combination, posts their reaction to the seed message. Time is incremented to the next time point and each group reads these newly-posted messages and computes their own optimal action-target combination by conditioning on the values in the message that had been directed towards them. These optimal action-target combinations are then posted to the bulletin board. When all groups have posted their action message(s) and the ecosystem submodel has posted updated expected values of its output nodes (the model's ecosystem metrics), the time variable is incremented to the next time point and this protocol is repeated.

This message posting protocol allows feedback loops through time to emerge without need for additional model structure. In particular, feedback from the ecosystem back to groups can occur through this protocol.

Forecasting

At each time step, each group selects a decision that they forecast will maximize their utility in the immediate future. This forecast is performed by taking into account what actions have just been directed at them and then, for each decision option, computing the expected utility (called here, their *overall goal attainment*) they would receive if they implemented that option. This forecast then, is maximum utility decision making conditional on the immediate past using the group's internal perceptions of how the world works.

Behavioural assumptions and decision making

- Political actors or *groups* interact with an ecosystem in a *political-ecological system.* Groups act to reach economic, militaristic, and political goals but hold internal, possibly distorted perceptions of other groups and the ecosystem. Groups interact with each other and with the ecosystem through time.
- A group's decision making and an ecosystem's responses are simulated with IDs. Each group is an agent. A separate ID contains the ecosystem submodel.

Learning

Submodel parameter values are not modified during the course of a simulation run. Hence, the simulator does not learn from past events. And, new decision options are not developed within submodels during a simulation run. Learning is restricted to (offline) statistical estimation of the model's parameters.

Population demography

All group submodels persist in the model. No new group submodels are added during a simulation run. All cheetah in the ecosystem submodel either die of old age or are shot. Cheetah can be born. These birth-death processes are stochastic.

Levels of randomness

Stochastic simulation is used to approximate the marginal probability distribution of each node in an ID and, from that, an approximate expected value of the `overall goal attainment` node under each combination of inputs and proposed decision option. Decisions are posted to the bulletin board with no further randomness. To avoid decision patterns that are artifacts of the order in which group IDs read the bulletin board, the order in which group IDs read the bulletin board is randomized each time step. The ecosystem submodel is a system of SDEs. Simulation is used to find an approximate solution to this system in the form of approximate expected values of the cheetah's birth rate, death rate, abundance, and abundance of its prey. These abundance values are posted to the bulletin board in the form of the fraction of the landscape over which cheetah (or herbivores) are detected. As abundance increases, this fraction increases.

Miscellaneous

Group IDs can issue up to two decisions per time step if there are at least two actions directed at them during the previous time step.

Functional Specification

Description of agents and other entities, action and interaction

The **id** modeling and analysis software system runs a JAVA executable form of this model. The following is a summary of descriptions of the decision making IDs that appear in [5, ch. 7].

President IDs

In this ID, (see Figure 5), the president has direct knowledge of the actions of the country's rural residents and pastoralists. The president's sole audience is the group of donors to his/her presidential campaign. The president's goals are to maintain political power and domestic order.

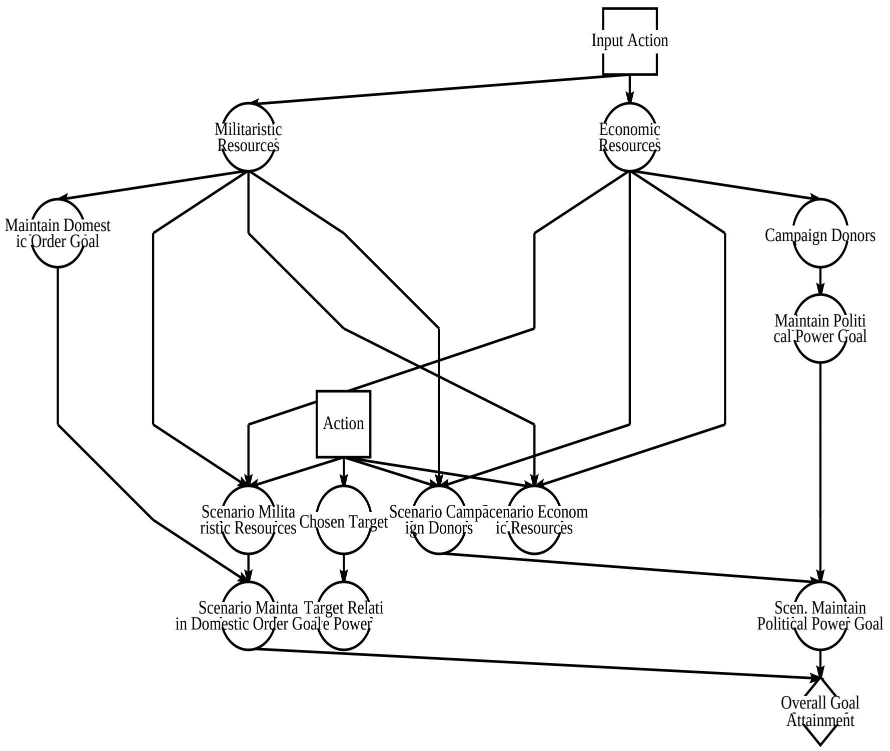

Fig 5. President ID. Square: decision options; Diamond: overall feeling of goal attainment. A decision (action) node along with the Situation Goal nodes affects Scenario Goal nodes.

Actions are derived from the East African cheetah EMT political actions data set.

Environmental protection agency IDs

EPA perceptions of the ecosystem's state are represented by herbivore prevalence and cheetah prevalence nodes, see Figure 6. These nodes are influenced by abundance values posted by the ecosystem ID. The `herbivore prevalence` node takes on the values *none*, *few*, and *many* and has a cumulative logit distribution with the one explanatory variable, `Herbivore Fraction Detected`. Values of this explanatory variable, are read from the bulletin board each time an EPA ID is solved. Likewise, `Cheetah Prevalence` is a cumulative logit chance node with the single explanatory variable being the ecosystem ID's computed value of `Cheetah Fraction Detected`.

The EPA's sole audience is the president. The goals of the EPA are to protect the environment, and to increase the agency's staff and budget.

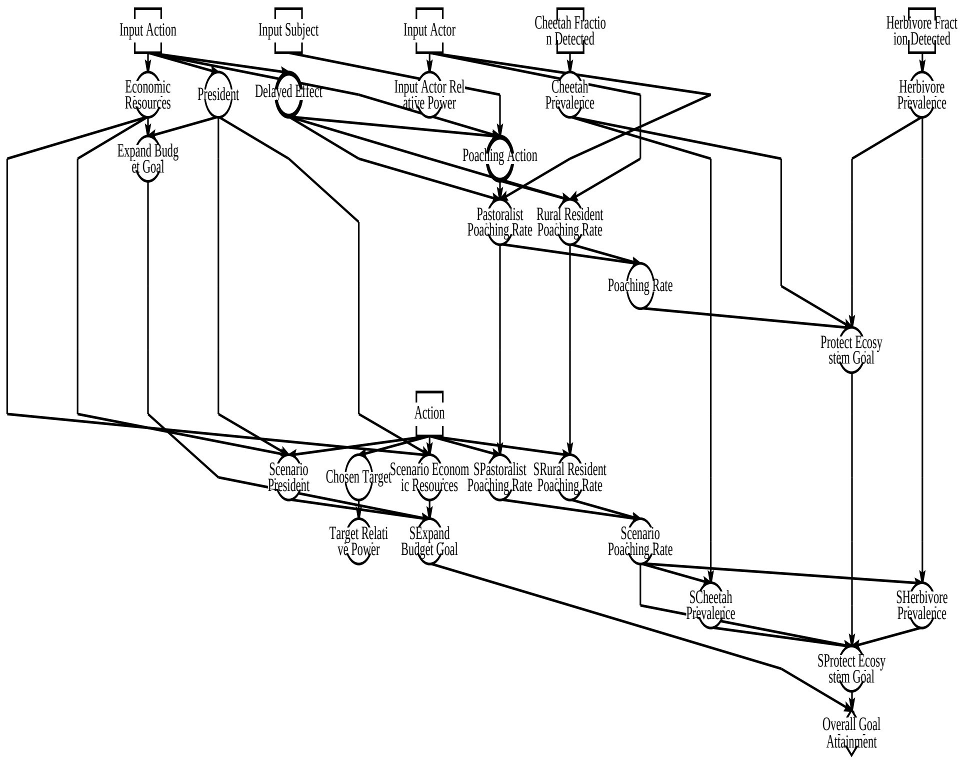

Fig 6. EPA ID.

Rural residents group IDs

A rural residents group ID has prevalence nodes for both herbivores and cheetah, see the EPA ID, above. Rural residents have the two conflicting goals of supporting their family, and avoiding prosecution (for poaching). See Figure 7.

A rural resident's sole audience is their family.

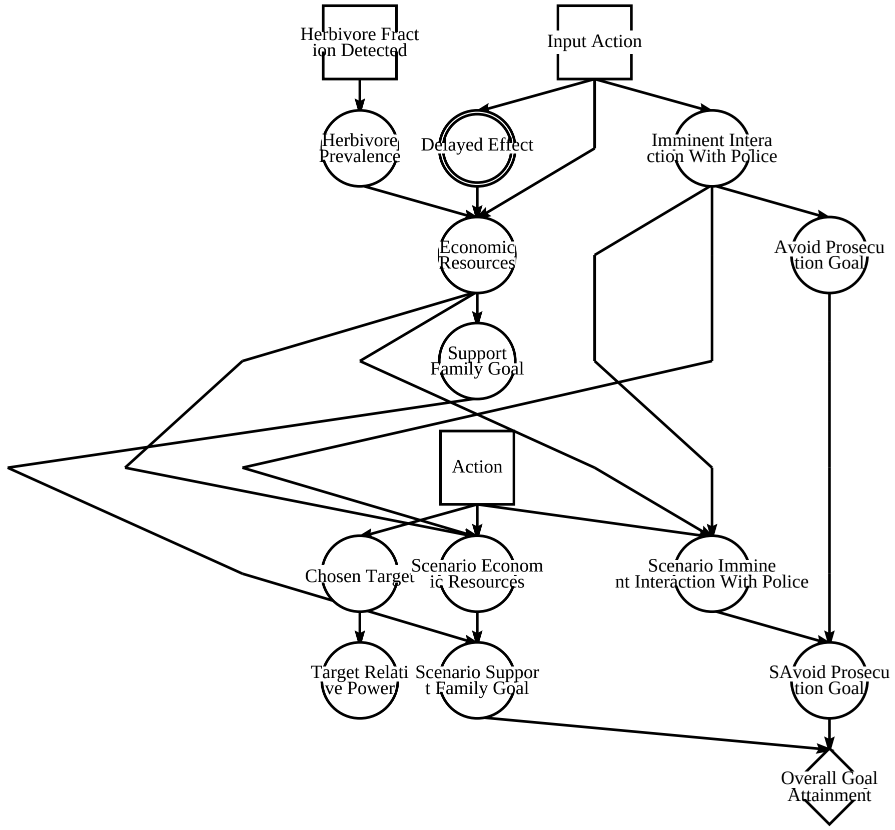

Fig 7. Rural residents group ID.

Pastoralists group IDs

Similar to the rural residents group ID, the pastoralists group ID has prevalence nodes for both herbivores and cheetah. Pastoralists have the three goals of supporting their family, protecting their livestock, and avoiding prosecution for poaching, see Figure 8.

A pastoralist's sole audience is their family.

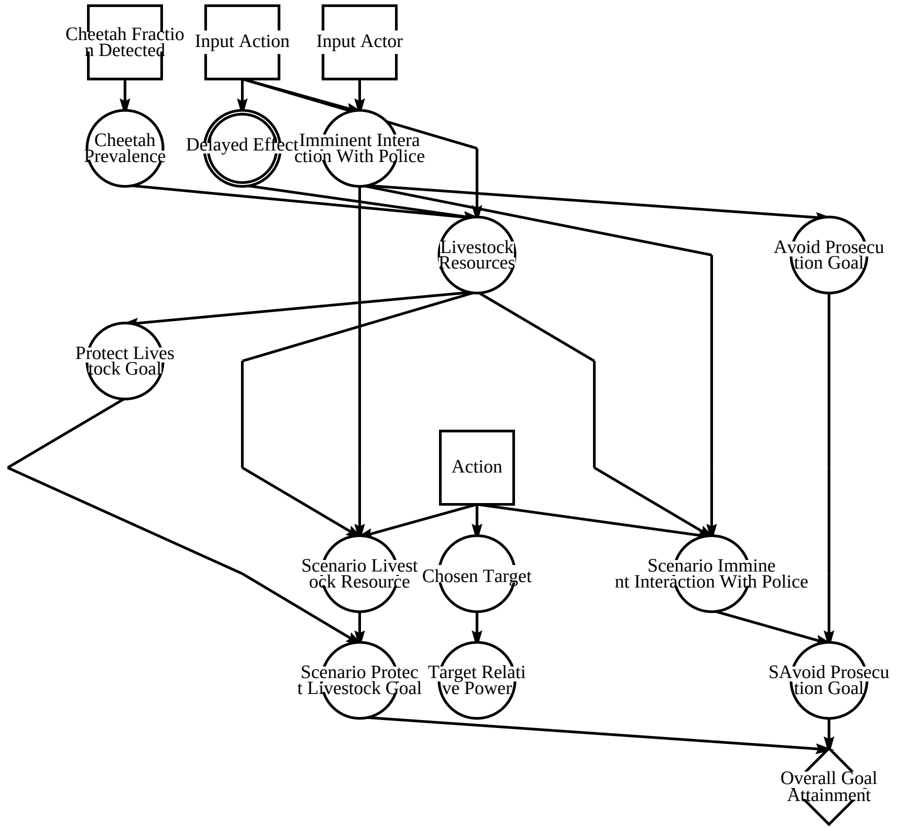

Fig 8. Pastoralists group ID.

Conservation NGOs group ID

Because this submodel is of a group of NGOs, an input action by a group in a particular country is assumed to be reacted to by an NGO in that same country. NGOs within this group keep track of input actions that affect wildlife in each country. These changes affect the NGO group's overall perceptions of cheetah and herbivore prevalence over the entire three-country area.

The audiences of NGOs are financial backers (donors) who reside in developed countries outside Africa, and the governments of the three host countries as embodied in each country's presidential office. See Figure 9.

NGOs have three goals: (1) conserve wildlife, (2) maintain productive relations with each host country's government, and (3) raise funds for their operations. Because an NGO's sole support is from external funds, input actions do not affect its budget. Rather, NGOs perceive that only the satisfaction level of external donors and the previous time step's economic resources affect its budget status.

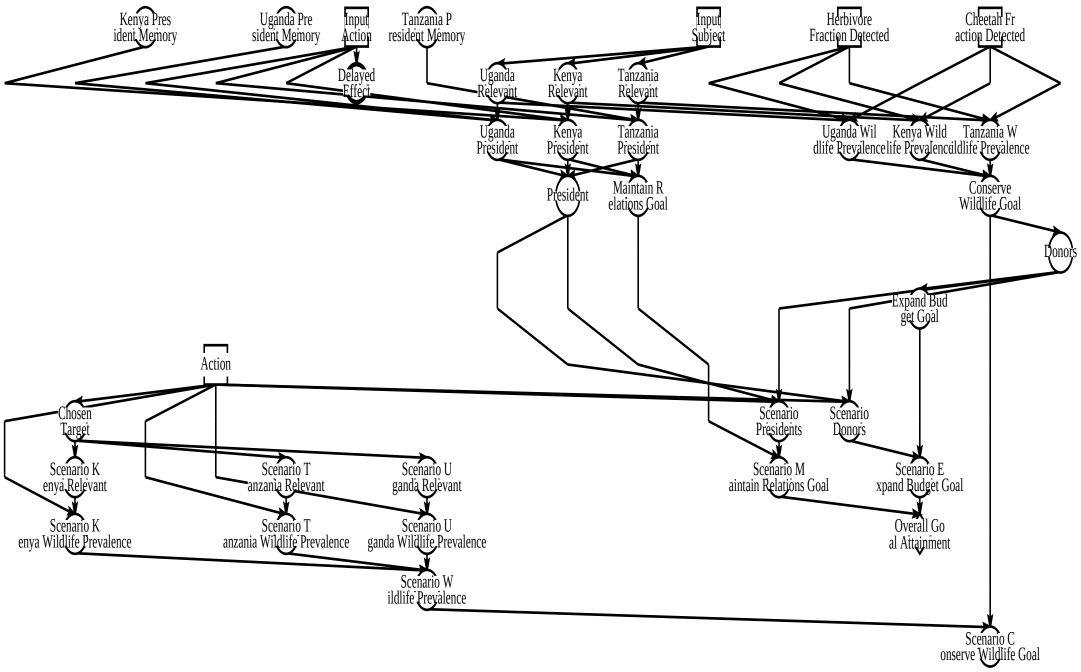

Fig 9. Conservation NGOs group ID.

Cheetah population dynamics submodel

Following the description in [5, pp. 109-119], the population dynamics of cheetah and their prey is modeled as a system of SDEs consisting of the dependent variables: birth rate (f_t), death rate (r_t), herbivore abundance (B_t), cheetah carrying capacity (K_t) and cheetah abundance (N_t). The ID that expresses this system of SDEs is built from five subIDs: inputs, habitat, direct effects on population dynamics, population dynamics, and observable random variables (see Figure 10).

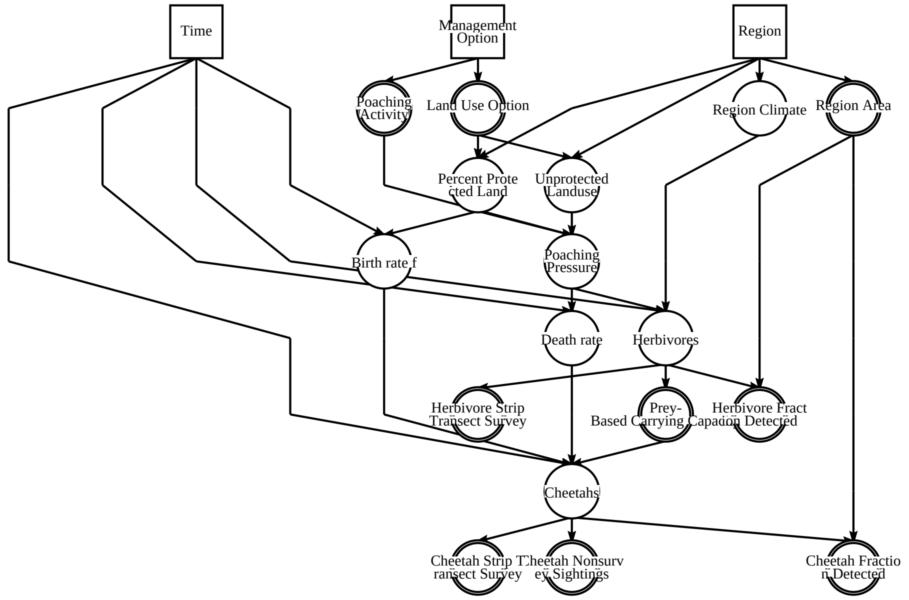

Fig 10. Cheetah-hosting ecosystem ID.

The herbivore abundance SDE is

$$\frac{dB_t}{dt} = \alpha_1 B_t(1 - B_t/\alpha_0) + \sigma dW_t \tag{1}$$

where α_0 is the habitat's herbivore carrying capacity, α_1 is the difference between herbivore birth and death rates, σ is the diffusion parameter, and W_t is a Wiener process. The initial value of B_0 is fixed at $0.6\alpha_0$.

The probability distribution of f_t at t is the solution to the SDE

$$df_t = -.5(\alpha_f + \beta_f^2(2f_t - 1))(1 - (2f_t - 1)^2)dt + .5\beta_f(1 - (2f_t - 1)^2)dW_t^{(f)}. \tag{2}$$

This SDE's solution is bounded between 0 and 1 making f_t a dimensionless, fractional birth rate (fraction of abundance). A similar development for the death rate leads to the SDE

$$dr_t = -.5(\alpha_r + \beta_r^2(2r_t - 1))(1 - (2r_t - 1)^2)dt + .5\beta_r(1 - (2r_t - 1)^2)dW_t^{(r)}. \tag{3}$$

Note that the birth rate decreases as α_f becomes increasingly positive and the death rate decreases as α_r becomes increasingly positive. The tendency of female cheetah to have litters within protected areas is represented by having the parameter α_f be conditional on the proportion of protected land in the region. Similarly, to represent the effect of poaching on the death rate, the parameter α_r is conditional on poaching pressure (H_t). Poaching pressure, in-turn, increases as (a) poaching activities increase, (b) the proportion of protected land decreases, and (c) the proportion of land set aside

for livestock increases. The variability of the sample paths of f_t and r_t is controlled by the parameters β_f and β_r, respectively.

Cheetah population dynamics is dependent on herbivore abundance through the cheetah carrying capacity variable, K_t. This relationship is modeled as a linear function: $K_t = \beta_0 + \beta_1 B_t$. Letting P, c, N_0 and β_N be fixed parameters, the cheetah abundance SDE is:

$$dN_t = \left[f_t(1 - P^{cN_t}) - r_t - (f_t - r_t)\frac{N_t}{K_t} \right] N_t dt + \beta_N dW_t^{(N)}. \quad (4)$$

The parameter P is the probability that a meeting does not result in a litter, the parameter c is the proportion of animals that meet over a short time period, the parameter N_0 is the initial population size, and the parameter β_N is the noise coefficient. All effects not explicitly represented (such as migration/emigration, and/or parameter values that are age-dependent) that could influence the within-region cheetah abundance differential (dN_t) are subsumed into the Wiener process differential ($dW_t^{(N)}$) term.

Initialization

Files

Each group submodel is specified with an ID definition file (`submodel_name.id`), and associated parameter value file (`submodel_name.par`). Each ID's parameter value file name is listed inside its ID definition file. A separate pair of these files is used to define the managed ecosystem. For example, for the managed ecosystem that hosts the East African cheetah meta-population, the files `ecosys.id` and `ecosys-hyp.par` are the cheetah population dynamics submodel definition file, and associated file of hypothesis-valued parameters, respectively. A simulator definition file (`simulator_name.id`) lists all of these individual submodel ID definition files.

Initializing actions and values

An initial action taken by one of the groups is specified in the simulator definition file. Initial cheetah population sizes are specified in the ecosystem ID's hypothesis-valued parameter file.

Action and interaction

See the Section: **Interaction protocols and information flows** for a description of how actions are generated and how the submodels interact with each other.

Run-time input

On a single PC, a run of the simulator is made by entering the command `idalone simulator-name.id` at the computer's command-line prompt.

References

1. Sanjay HA, Vadhiyar SS. A strategy for scheduling tightly coupled parallel applications on clusters. Concurrency and computation: Practice and experience. 2009;21: 2491-2517.

2. SDSC. TSCC Quick Start Guide. San Diego Supercomputer Center. 2018. Available from `http://www.sdsc.edu/support/user_guides/tscc-quick-start.html`

3. Adaptive Computing. 6.3 Fairshare, Maui Schedule Documentation. 2018. Available from
`http://docs.adaptivecomputing.com/maui/6.3fairshare.php`

4. Wolf S, Bouchaud J, Cecconi F, Cincotti S, Dawid H, Gintis H, van der Hoog S, Jaeger CC, Kovalevsky DV, Mandel A, Paroussos L. Describing economic agent-based models – Dahlem ABM documentation guidelines. Complexity Economics. 2013;2: 63-74.

5. Haas TC. Improving natural resource management: Ecological and political models. Chichester, U.K.: Wiley-Blackwell; 2011.
