# Supplementary material for: Developing political-ecological theory: The need for many-task computing
Source: PLoS One. 2020 Nov 24;15(11):e0226861. doi: 10.1371/journal.pone.0226861 (PMC7685461; doi:10.1371/journal.pone.0226861)
Supplement: Attachment — Submitted filename: rev2r0rspns.pdf [file pone.0226861.s003.pdf]

Response to Reviewers

Note: Below, reviewer comments are numbered. These numbers appear in the "Revised Manuscript with Track Changes" in the form: "Comment xx:" where xx is the comment number.

Reviewer #2 Comments:

1. The article is focused on developing political-ecological theory. To this purpose, it considers that models should be better analysed in depth in order to increase their credibility. This comes with a huge need of High Computing Performance. Authors thus propose to use Many-Task Computing coded with JavaSpacesTM.

2. The major critics again this article is that it considers many parts (especially in Section 1 and 2), that are related to the subject, but not directly to the contribution of the article. This makes the article too long and a little bit confused on its main objective. As an example all the part about agent-based modeling can be omitted (or reduced a lot). Similarly does the presentation of all the statistical tools really need to be so detailled, as the main question of the paper is on parallelisation of the computation?

> I believe that an overview of how my formulation of agent-based modeling of social group decision making is needed to have the reader appreciate why these models require so many parameters and why they require such large amounts of computing time to run and to be statistically analyzed.
>
> I believe that Section 2's in-depth description of the mathematical foundations of the algorithms that my EMT implements is a strength of the manuscript and justifies my statement that my EMT should be the new benchmark for establishing the credibility or lack thereof of a simulable stochastic model. I also believe that a complete mathematical treatment of statistical parameter estimation as opposed to model calibration for instance, can only be made clear if all relevant equations are presented. The same goes for critic-driven sensitivity analysis, and the search for politically feasible ecosystem management policies. Please see lines 363-371 and the remainder of these Responses to the Reviewer for further justification for why such a level of mathematical detail is necessary.

3. In contrarily, the choice of the Many-Task Computing approach and coded with JavaSpacesTM is not supported by a state of the art of the existing alternatives approaches.

   This comment is a special case of comment 12. Please see my response therein.

4. l31: "There is a need to acknowledge the complexity of political-ecological systems" There is not a need anymore to acknowledge the complexity of political-ecological systems, t

   The Macmillan dictionary includes the following as a definition of the word *acknowledge*: "to know or recognize that someone or something is important" (Macmillan 2020).

   My statement uses the word in this way. If it were common knowledge that political-ecological systems are complex, one would not see statements announcing that political-ecological systems are complex since such a fact would have been previously accepted by the scientific community. Therefore, one way to determine if there is no need to acknowledge that political-ecological systems are complex, is to search the current scientific literature for statements that declare that there is a need to acknowledge that political-ecological systems are complex. If none are found then one could conclude that there is no need to acknowledge that political-ecological systems are complex because no one is saying there is such a need. I performed such a search. First, I found that the journal *Ecological Modelling* is concerned enough about the lack of integrated, i.e., complex socio-ecological models, that they include this sentence in their Guide to Authors: "There is rapid rise in the need for integrated socio-ecological models which fall within the journal scope, but the focus must be on the ecological aspects not the sociological or economic." (Elsevier 2020).

   Then, I discovered Dressel et al. (2018) noting that "To balance societal needs and ecological functions, the complex interactions between humans, wildlife, and habitats must be fully understood Apollonio et al. (2017)."

Even without further research, one could conclude at this point that although several articles do indeed acknowledge the complexity of political-ecological systems, several authors continue to see a need to make the point that such systems are complex. In other words, these authors see a need to acknowledge that political-ecological systems are complex. Therefore, I don't see my statement as unnecessary and hence would prefer to leave it in my manuscript in order to help motivate my main message that the conservation community needs to focus more attention on building credible models of political-ecological systems and realize that doing so will require new techniques because these systems are complex.

See lines 31-32.

5. l54 : "Unless such a model is shown to be credible using in-part, appropriate statistical methods, any "recommendations based on output from the model may receive only mixed acceptance by those affected." This has been showed that, even if the model is well-calibrated and analysed, it can be not been accepted by decision-makers, in particular due to a lack on involvement (see articles of A. Smagl) or that many models are accepted even if not realistic or calibrated (cf. all the works in participatory approaches and participative simulation).

    This reviewer comment claims that a model's acceptability to decision makers is not about how credible the model is as defined in my manuscript but rather how the model came to be known to the decision maker. Specifically, if the decision maker was involved in the model's development, the decision maker will accept the model – even if the model's agreement with data is poor and/or the model contains excessively sensitive parameters. The reviewer mentions papers written by Alexander Smajgl to support this view of model acceptance.

    This comment is implicitly referencing the idea of *post-modernist science* or equivalently, *post-normal science.* This conceptualization of "science" is in contrast to *experimental science.* An experimental science such as physics, chemistry, or genetics relies on the outcome of experiments to judge the credibility of a model. To quote a recent theoretical scientist who produced

a number of significant contributions to physics:

> It doesn't matter how beautiful your theory is, it doesn't matter how smart you are. If it doesn't agree with experiment, it's wrong.

Richard P. Feynman (Natural History Museum 2020).

Indeed, the most famous recent example of this is the gradual acceptance of quantum mechanics due to repeated experiments by different scientists trying to prove that their rival scientists were wrong (O'Connor and Robertson 1996).

On the other hand, post-normal science accepts non-experimental ideas to contribute to the understanding of a phenomenon. For instance, Roberts (2005) states

> Knowledge is the product of the social construction of the interaction of people involved in a common situation or endeavor. From an epistemological perspective, participatory models grow out of a postmodernist view of science in which the concept of truth or knowledge is a socially constructed event.

Based on earlier work, Smajgl and Ward (2015) offer an approach to model construction they claim will lead decision makers to accept the model and use it to guide ecosystem management policy. They call this approach, *participatory modeling.* These authors apply participatory modeling to the political-ecological system surrounding water management in the Greater Mekong Region in Vietnam. In their description of how they built the model through a series of workshops, these authors note that

> ...the modeling is not used as a predictive tool, presenting emerging patterns as alternative heuristics and not as forecasts.

This participatory model then, according to Smajgl and Ward, is not intended to simulate the effect of different management policies on the Greater Mekong Region. It is not clear then, how a participatory model is to be used to inform ecosystem management policymaking.

By way of contrast, the ecosystem management tool (EMT) that I describe in my manuscript provides a systematic methodology for building a credible model of a political-ecological system that, because of its forecasting capability, can be used to evaluate the effects of proposed ecosystem management policies.

In a section that explains the theoretical underpinnings of participatory modeling, Smajgl and Ward (2015) declare that

> ...decision makers construct their own heuristics guiding their decision making in a constructed reality ... participatory framework combines future visions articulated by participants with the provision of scientific evidence specific to the research focus identified by national decision makers during the scoping study. ...Constructivism shifts the 'support of multiple perspectives or interpretations of reality, knowledge construction, and context-rich, experience-based activities' to the centre of learning processes. ... The theoretical underpinnings of the research assumes that people construct their version of reality, that many system dynamics are non-linear and complex and that emergent phenomena can differ substantially from the predicted outcomes of linear interactions. Stearman (2008) contends that people are cognitively too constrained to comprehend complex problems, forcing people to develop simplifying heuristics.

Apparently, participatory modeling accepts the view that there is more than one reality. This is contrary to experimental science wherein the assumption is that there is a single reality. And further, it appears that participatory modeling is founded on the idea that because people cannot comprehend complex models, for policymaking purposes, such models should be replaced with heuristics that people can easily understand. Again, an experimental science has as its goal, the development of a model of some phenomenon that, however complex the model might be, is a comprehen-

sive representation of the phenomenon that is able to produce predictions that exhibit an error rate against real-world observations that is lower than that of blind guessing.

Smajgl and Ward (2015) include references to Smajgl (2010) and Smajgl (2013a). Neither of these articles nor Smajgl and Ward (2015) contain any content concerning the possibility that decision makers will reject any model based on experimental science unless they, the decision makers participated in its development. It is not clear then, what scholarship is being referred to when the statement is made in the reviewer comment that "This has been showed that, even if the model is well-calibrated and analysed, it can be not been accepted by decision-makers, in particular due to a lack on involvement (see articles of A. Smagl)..."

Next, I discovered that Röckmann et al. (2012) define participatory modeling as follows.

> To conclude, participatory modelling has the potential to facilitate and structure discussions between scientists and stakeholders about uncertainties and the quality of the knowledge base; it can contribute to collective learning, increase legitimacy, and advance scientific understanding. However, when approaching real life problems, (participatory) modelling should not be seen as the priority objective. Rather, the crucial step in a science-stakeholder collaboration is the joint problem framing in an open, transparent way, in order to ensure that scientists tackle the relevant problems. Where people communicate with each other, it improves people's ability to understand each other.

Hence, these authors, similar to Smajgl and Ward (2015) do not see participatory modeling as a modeling methodology that replaces models based on experimental science, rather, the shared view is that participatory modeling should be used to delineate areas of uncertainty and foster communication between stakeholders (including decision makers) and scientists engaged in

building positivist computer models.

Röckmann et al. (2012) further comment:

> A policy situation can be considered post-normal (science) when stakes are high and scientific knowledge is uncertain which often is the case for fisheries. In such situations, one cannot rely on textbook knowledge, and trust that scientists alone will be able to give the answers - because there is not one single answer due to the uncertainties and decision stakes involved. The different types of uncertainties have traditionally been dealt with insufficiently by the science, and some scientists have advocated to bring them to the centre of the policy debate. A central element of post-normal science is extended peer review, where the scientific "peer review community" is extended to include stakeholders. An extended peer review process extends beyond simply ensuring the scientific credibility of results to ensuring the relevance of the results for the policy process. Crucial for an extended peer review is that non-experts understand the implied uncertainties in scientific knowledge so that management actions can take them into account.

This concern about bringing uncertainties to the forefront of policymaking is why my EMT delivers detailed estimates of parameter uncertainty and sensitivity that are based on comparisons of model output with data. It is essential that any uncertainty analysis be conducted with established statistical procedures that are data-based. Otherwise, the uncertainty analysis is not credible in the eyes of those it is intended to inform: stakeholders who will be affected by ecosystem policy decisions that are suggested by EMT outputs.

The position taken in my manuscript is that the way to make science relevant to ecosystem management is to develop a credible model of the attendant political-ecological system. To be credible, this model needs to be

fitted to data with established statistical methods and needs to be stochastic so that all sources of uncertainty are explicitly present in the model. This approach to policy-relevant science is an alternative to post-normal science and is distinctly a positivist, experiment-based approach to informing ecosystem management decisions. In this sense, my EMT addresses the need to educate stakeholders about the policy-relevant model and to present to them a credible model whose sources of uncertainty are clear. This highlighting of model uncertainties is the EMT's answer to the focus on uncertainty that is a hallmark of participatory modeling.

In addition, this focus on maturing the science of political-ecological systems is similar to a direction being taken in ecology itself: assessing the skill of large ecosystem models. For instance, Olsen et al. (2016) declare "We show that it is possible to not only assess the skill of a complicated marine ecosystem model, but that it is necessary do so to instill confidence in model results and encourage their use for strategic management."

Of course, it is imperative that decision makers use models to inform ecosystem management decisions. It is is counter-productive, however, to advance a modeling methodology that has the potential to produce a policy-guiding model whose outputs do not agree with data – no matter how acceptable the model might be to decision makers. This is because such a model, having gotten a cause and effect relationship in the ecosystem wrong, might produce recommendations that, if implemented could lead to the opposite of the anticipated effect. Participatory modeling is in-effect, willing to trade model agreement with data for a socially-inclusive model development exercise that results in a model that is based on easy-to-understand mechanisms. My EMT on the other hand, builds credibility of the model in the eyes of a decision maker through demonstrations of the model's agreement with observations and its insensitivity to minor misspecification of its parameters. This approach is in alignment with the direction that I think the science of political-ecological systems should be taking: becoming an

experimental science rather than a post-normal science. This focus is now explicitly stated on lines 63-97.

6. l135: "A literature search uncovered only two articles describing the statistical estimation of a socio-ecological model's parameters, namely, [23], and [17]." What about: "Calibration of simulation platforms including highly interweaved processes: the MAELIA multi-agent platform. June 2014, Conference: 7th Intl. Congress on Env. Modelling and Software At: San Diego, CA, USA, Romain Lardy et al. "

   In Lardy et al. (2014), randomness is coming from a finite set of parameter values that lie on the Pareto optimal front that represents all non-dominated solutions to the multi-objective weight least squares parameter estimation solution across all possible weightings of the multiple objective functions (Lardy et al. 2014, Figure 2). In statistical parameter estimation as employed in my EMT, parameter uncertainty is driven by the sampling distribution, i.e., the variability in the data collection activity causes each parameter to have a parameter estimate distribution. Hence, the boxplots of parameter uncertainty given in in Lardy et al. (2014) refer to parameter estimate variability caused by an analyst choosing different sets of weights for the multiple objective functions. This source of uncertainty has nothing to do with the uncertainty caused by sampling from a population defined by the observable variables of a political-ecological model. Hence, Lardy et al. (2014) does not provide an example of statistical estimation of a political-ecological model. This is a good example of why the technical details of a statistical assessment of a model need to be made explicit. Hill (1998) gives the following definition of model calibration: "In model calibration, various parts of the model, including the value of model input values, are changed so that the measured values (often called observations) are matched by equivalent simulated values, and, hopefully, the resulting model accurately represents important aspects of the actual system." Note that calibration is not statistical parameter estimation. The latter activity produces confidence intervals for the population values of the parameters while the former does not.

7. In addition, it would be surprising that in all the models of socio-environmental systems (being agent-based or not), there is no parameter estimation...

   Note that in lines 171-172, I report that I searched for articles describing *statistical* parameter estimation of political-ecological models – and found only two. I do report, however, on a number of articles that statistical estimate the parameters of either a strictly social model or a strictly ecological model. This result is a consequence of the formidable and new challenge facing the scientific community: Statistically estimating the parameters of an integrated model of political and ecological processes. Referring to my response to reviewer comment 4, above, this challenge reinforces why there is a need to acknowledge the complexity of political-ecological systems and lends an urgency to the need for advances in this area such as those delivered in my manuscript.

8. Finally, the issue of sensitivity analysis, calibration, parameter estimation ... could be tackled more generally than only in the context of SES systems, but also in any other applications (e.g. urban context, cf Denise Pumain & Romain Reuillon. Urban Dynamics and Simulation Models.)

   Pumain and Reuillon (2017) give a genetic algorithm method for calibrating their agent-based model of the growth of a cluster of small human settlements into an urban center. And, they provide a sensitivity analysis method that is also based on a genetic algorithm. These authors acknowledge the heavy computational demands of these two algorithms when applied to the evolution of settlements into cities. To address this concern, similar to the MTC paradigm implemented in my manuscript, these authors develop a software package for running their algorithms on a cluster computer.

   Yes, my EMT could indeed be applied to building models of settlement-to-city evolution. And doing so would result in models that would possess more credibility than can be achieved with the methods given in Pumain and Reuillon (2017). This is because my EMT is based on a stronger mathematical foundation, takes a statistical approach to parameter estimation

(see my response to the previous reviewer comment), provides critic-driven sensitivity analysis, and is implemented in a write-once – run-everywhere software architecture based on MTC principles.

I agree that my EMT could be used to develop more credible agent-based models in fields other than political-ecological systems such as epidemiology, information diffusion in social systems, and economics.

See lines 1081-1086.

9. p8: "A political-ecological system simulator (hereafter simulator) is an executable computer program capable of approximating the outputs of a stochastic model of a political-ecological system." This definition can be applied in many cases of models. But in the case of agent-based models (that is the kind of models that is presented in this paper), defining it only related to its outputs is limitative, as its main strength relies on its capability to represent and understand inner dynamics. To fit with outputs, modern AI models are often more accurate.

   I appreciate the reviewer making this point. I now mention that artificial intelligence (AI) models that are stochastic in their outputs can be included in this definition such as the stochastic neural network model of Ling et al. (2016).

   See lines 251-254.

10. l215: this is only a technical point of view that occults all the social acceptability and power game that are important in model adoption.

   Rather than a description of a technical procedure for model goodness of fit, my EMT is a comprehensive approach to building credible models of political-ecological systems whose outputs can suggest ecosystem policies that, because of the EMT's delivery of a credible model, will be accepted by all stakeholders in the managed ecosystem. A part of this EMT is a very technical procedure for fitting the model to data and assessing its credibility through statistical measures of uncertainty and a policy-relevant analysis of its sensitivity to parameter misspecification. These technical details are the foundation on which my EMT rests and are therefore, the most salient

parts of my manuscript. I interpret this comment as a continuation of comment #5, above and hence my responses therein are relevant here.

11. l228: ”EMT proceudre”

    - how is it related to the idea of modeling methodolofy ?

    - Step 1 : what does the idea of boundaries cover ? is it spatial, temporal ? thematic ?

    - Do we need to specialize an ecosystems to one with endanger species ? Why does this approach does not aim at more generality ?

    - the various steps of the procedure have very different level of precision: there are details about files on one side (very low and technical level) and other parts are really higher level (e.g. identify the ecosystem ...)

    My EMT is a procedure for building an agent-based model of a political-ecological system and performing the three steps needed to use that model to manage that ecosystem. These steps are to first, statistically estimate the parameters of the model, second, assess the model's credibility, and only then, use the model's outputs to identify effective and politically feasible ecosystem management policies.

    My EMT's Step 1 pertains to the spatial boundaries of the ecosystem to be managed, see lines 271-272.

    I focus on endangered species in my EMT because I am submitting my manuscript to the 2020 PLOS Collection on Biodiversity Conservation. I do acknowledge that my EMT could be used to manage ecosystems that are under other pressures such as soil contamination with heavy metals, or freshwater pollution through agricultural runoff. See lines 1076-1078.

    An effective plan includes the necessary detail at all relevant scales so that individuals attempting to implement the plan may proceed without having to stop along the way and invent protocols that the original plan did not cover (Przbyla 2020, Chen et al 2006). For instance, because my EMT has different research groups developing different submodels, it is important to maintain these submodels in different files so that these modeling

activities can proceed asynchronously and yet allow the resulting submodels to be integrated to form the complete model of the political-ecological system under study. The challenge of coordinating several collaborating teams on a project is addressed in Chen et al. (2006). Chen et al. (2006) describe their *collaboration project management framework* (CPMF). One component of CPMF is *comprehensive knowledge capture.* This consists of a project repository that contains all documents pertinent to the project. The authors note that a limitation of their CPMF is that it lacks a "detailed implementation system architecture." Clearly, these authors view a complete project management system as having a detailed protocol of what documents (files) each collaborating team member is responsible for producing and exactly where these documents will be stored. My EMT can be viewed as a project management system but with extensions for statistical analysis, sensitivity analysis, and evaluation of proposed policies. Hence, to use my EMT, protocols are needed that span several scales from the fine detail of what files will be shared between groups up through the single set of boundaries that define the ecosystem to be managed.

12. As parallelisation seems to be the key point of the paper a State of the Art about parallelization technics and MTC would be necessary. In particular, only JavaNameSpace and MTC are presented and no other alternatives.

Actually, my manuscript evaluates the main alternative to a JavaSpaces implementation of a MTC program: the so-called message passing interface (MPI), see lines 733-776. In this evaluation, it is found that MPI abstracts program control at too low a level for effective control of a MTC program. In addition, an MPI program, unlike JavaSpaces, cannot be conveniently run on a range of devices. See lines 824-830.

Note that MTC is not a parallesization technique. Rather, it is a paradigm for organizing the cooperation between several large, semi-autonomous programs. These programs may run simultaneously or sequentially. Parallelization on the other hand, is a much more low-level computing concept

in that it concerns how an algorithm (small program) can be modified so that it can be executed by several processors running simultaneously wherein each processor executes some portion of the needed computations while passing and receiving a certain number of messages to and from the other processors.

13. l1000 : The conclusion that taking more land to build a reserve with a low resistance to its implementation seems unrealistic. This would requiers more details.

    The reviewer has an excellent point. I have added the needed additional explanation for this counter-intuitive result. Specifically, in a recent paper, Abukari and Mwalyosi (2020) use survey data collected from residents living close to protected areas in Ghana and Tanzania to reach their conclusion that local people will find a protected area advantageous to their livelihoods if they are included as equal participants in decisions concerning the management of the protected area and, for nonpastoralists, if there is land outside the protected area where they can grow crops. I now state that the MPEMP will meet low resistance only of the above two conditions are part of its implementation.

    See lines 1055-1064.

References

Abukari, H. and Mwalyosi, R. B. (2020), "Local Communities' Perception about the Impact of Protected Areas on Livelihoods and Community Development," *Global Ecology and Conservation,* 22, doi: 10.1016/j.gecco.2020.e00909.

Chen, F., Romano, Jr., N. C., Nunamaker, Jr., J. F. (2006), "A Collaborative Project Management Approach and a Framework for Its Supporting Systems," *Journal of International Technology and Information Management,* 15(2): 1-15. Retrieved March 9, 2020, from `https://core.ac.uk/download/pdf/55335547.pdf`

Dressel, S., Ericsson, G., and Sandström, C. (2018), "Mapping Social-Ecological Systems to Understand the Challenges Underlying Wildlife Management," *Environmental Science and Policy,* 84: 105-112.

Elsevier (2020), *Ecological Modelling: Guide for Authors.* `https://www.elsevier.com/journals/ecological-modelling/0304-3800/guide-for-authors`

Hill, M. C. (1998), *Methods and Guidelines for Effective Model Calibration,* Report 98-4005, U.S. Geological Survey, Denver, CO. inside.mines.edu/~epoeter/583CSM/EffectiveCalibration_WRIR98-4005.pdf

Ling H., Samarasinghe S., Kulasiri D. (2016) Stochastic Neural Networks for Modelling Random Processes from Observed Data. In: Shanmuganathan S., Samarasinghe S. (eds) Artificial Neural Network Modelling. Studies in Computational Intelligence, vol 628. Springer. doi: https://doi.org/10.1007/978-3-319-28495-8_5

Macmillan (2020), Definition of *acknowledge*. Retrieved March 5, 2020, from https://www.macmillandictionary.com/us/dictionary/american/acknowledge

Natural History Museum, (2020), *Nature of Science terms*, Natural History Museum, London. Retrieved March 5, 2020, from https://www.nhm.ac.uk/content/dam/nhmwww/about-us/visitor-research/nature-of-science-terms.pdf

O'Connor, J. J. and Robertson, E. F. (1996), *A History of Quantum Mechanics,* School of Mathematics and Statistics, University of St Andrews, Scotland. Retrieved March 5, 2020, from mathshistory.st-andrews.ac.uk/HistTopics/The_Quantum_age_begins.html

Olsen, E., Fay, G., Gaichas, S., Gamble, R., Lucey, S., and Link, J. S. (2016), "Ecosystem Model Skill Assessment. Yes We Can!" *PLOS ONE*, 11(1): e0146467. doi:10.1371/journal.pone.0146467

Przbyla, J. (2020), *The Devil is in the Details (or, Why a Detailed Project Plan is Necessary), Woolpert*. Retrieved March 9, 2020, from https://woolpert.com/resource/devil-details-detailed-project-plan-necessary-2/

Pumain, D. and Reuillon, R. (2017), *Urban Dynamics and Simulation Models,* New York: Springer-Verlag. Retrieved March 23, 2020, from https://openmole.org/all/8.0-RC1/paper/urbanDynamics.pdf

Roberts, R. N., (2005) "Community: The Ties That Bind," (in) *Culture & Context in Human Behavior Change: Theory, Research, and Applications,* (eds.) C. R. O'Donnell and L. A. Yamauchi, New YorK: Peter Lang Publishing, 278 pages.

Röckmann, C. et al. (2012), "The Added Value of Participatory Modelling in Fisheries Management – What has Been Learnt?" *Marine Policy*, 36(5): 1072-1085.

Smajgl, A. and Ward, J. (2015), "Evaluating Participatory Research: Framework, Methods and Implementation Results," *Journal of Environmental Management,* 157: 311-319.
