# Supplementary material for: Developing political-ecological theory: The need for many-task computing
Source: PLoS One. 2020 Nov 24;15(11):e0226861. doi: 10.1371/journal.pone.0226861 (PMC7685461; doi:10.1371/journal.pone.0226861)
Supplement: Attachment — Submitted filename: r2rspns.pdf [file pone.0226861.s004.pdf]

Response to Reviewers

Academic Editor Comments:

1. **In summary, it has been repeatedly recommended that the manuscript be significantly reduced in length, providing a clearer, tighter focus on its contribution, and allowing readers to more easily engage with, and make use of, the ideas presented.**

   I have significantly shortened the manuscript. It is now 12,994 words. To get some idea of an appropriate length, I selected the following five PLoS ONE articles and found them to contain 18,775, 7,906, 13,424, 11,101, and 10,995 words, respectively.

   - Bocci M, Sangiuliano SJ, Sarretta A, Ansong JO, Buchanan B, Kafas A, et al. (2019) Multi-use of the sea: A wide array of opportunities from site-specific cases across Europe. PLoS ONE 14(4): e0215010.
   - Campbell BW, Marrs FW, Böhmelt T, Fosdick BK, Cranmer SJ (2019) Latent influence networks in global environmental politics. PLoS ONE 14(3): e0213284.
   - Ling T, He Y (2020) The remanufacturing evaluation for feasibility and comprehensive benefit of retired grinding machine. PLoS ONE 15(6): e0234603.
   - Olsen E, Fay G, Gaichas S, Gamble R, Lucey S, Link JS. (2016) Ecosystem model skill assessment. Yes we can! PLoS ONE, 11(1): e0146467.
   - You C, Lin DKJ, Young SS (2018) Time series smoother for effect detection. PLoS ONE 13(4): e0195360.

   I now believe my manuscript is as long as a typical PLoS ONE article. I would be happy, however, to further shorten it if it is otherwise found acceptable.

   I have also focused the paper on what I believe its key contributions are.

2. **In addition, it is recommended that you take note of, and address, comments regarding computational validation presented, and the need for a broader consideration of possible approaches to achieving parallel implementation.**

The paper now contains additional discussion and results regarding the validity of its computational methods, see lines 805-811. Also, I now point out that at least three alternatives to JavaSpaces exist, see lines 516-528. I make it clear that these alternatives can deliver the parallel computations described in Section 2.

Reviewer #3 Comments:

1. **In its current form, the manuscript is unpublishable. I say this not unkindly, but constructively. The manuscript reads far more like a thesis, but not one that would be passable. The manuscript is far too long and contains a lot of irrelevant material that obscures its contributions (the important bits). I believe that contributions are there, but they are completely obscured.**

   I have significantly shortened the manuscript. It is now 12,994 words. To get some idea of an appropriate length, I selected the following five PLoS ONE articles and found them to contain 18,775, 7,906, 13,424, 11,101, and 10,995 words, respectively.

   - Bocci M, Sangiuliano SJ, Sarretta A, Ansong JO, Buchanan B, Kafas A, et al. (2019) Multi-use of the sea: A wide array of opportunities from site-specific cases across Europe. PLoS ONE 14(4): e0215010.
   - Campbell BW, Marrs FW, Böhmelt T, Fosdick BK, Cranmer SJ (2019) Latent influence networks in global environmental politics. PLoS ONE 14(3): e0213284.
   - Ling T, He Y (2020) The remanufacturing evaluation for feasibility and comprehensive benefit of retired grinding machine. PLoS ONE 15(6): e0234603.
   - Olsen E, Fay G, Gaichas S, Gamble R, Lucey S, Link JS. (2016) Ecosystem model skill assessment. Yes we can! PLoS ONE, 11(1): e0146467.
   - You C, Lin DKJ, Young SS (2018) Time series smoother for effect detection. PLoS ONE 13(4): e0195360.

   I now believe my manuscript is as long as a typical PLoS ONE article. I would be happy, however, to shorten it further if requested.

2. **The emphasis is all wrong. The manuscript is to much front loaded particularly in the introduction and literature review sections. These need to short and sharp and really drive the motivation of the novel contribution of the work. At the moment they are bloated and contain so much discussion that the reader easily forgets what the paper is about.**

I have achieved this reduction in length mainly by focusing the paper on what I see as its key contributions. To this end, I've removed some of the more philosophical material in Sections 1 and 2.

3. **Similarly, the important back-end is very scant. There needs to be a much richer discussion of results, the summary of the work and particularly what the future work can be.**

   I have added more discussion of the computational aspects of my EMT procedure, see lines 767-775, 790-797, and 805-811. I have also commented on what future work is needed, see lines 830-845.

4. **In terms of the front-end, why do you have all these very large quotes? They add nothing to the paper. Remove them all and just paraphrase if necessary.**

   I have replaced all of the lengthy quotes with paraphrasing.

5. **Section 1.3: A new algorithm (step-by-step procedure) is added in the literature review section. This would need to go later in the paper.**

   I have moved the EMT procedure forward to the Materials and Methods section.

6. **In general, please make algorithms short with minimal explanation each step, otherwise they become difficult to read. If you need more explanation, provide it in the body of the text.**

   I have removed some text from the EMT procedure and the confidence interval algorithm, see lines 243-290, and 386-395. I have also shortened the sensitivity analysis algorithm, see lines 460-484.

7. **There is a Figure 2 caption, but no actual figure.**

   I have verified that all Figures appear after the submission's text in the document prepared by the *PLoS One* submission system.

8. **As intimated above, more extensive computational evidence would be good.**

   I have added additional computational evidence, see lines 805-811.

9. **Try to read some other PLOS ONE papers to see the form of them. I'm sure you'll find this helpful when you are revising your manuscript.**

   I have done so and have attempted to emulate their succinctness.

Reviewer #4 Comments:

1. **this task would have been made significantly easier if the track changes version had been produced used latexdiff (https://ctan.org/pkg/latexdiff) instead of manual bolding of content. While the Comment n markers were helpful in linking sometimes modified, typically additional, content to the relevant comments, it was impossible to see if content was deleted or otherwise edited without going through both versions side-by-side. This showed that some of the bolded content has not been modified while some other changes have been made that have not been marked at all.**

   I have used `latexdiff` to prepare the Revised Manuscript with Track Changes.

2. **It is clear, from the detail in the manuscript and the responses to the original reviewer's comments, that the author is passionate about the work and about making it technically sound. I appreciate the additions to the work have been made to address some of that reviewer's comments. However, the original reviewer clearly identified that the work was too long, with many details that could have been reduced or omitted (because they are covered elsewhere in the literature) and this revised version is even longer, at the upper end of a typical Honours (2-semester research) thesis. Based on my reading I am compelled to agree.**

   I have significantly shortened the manuscript. It is now 12,994 words. To get some idea of an appropriate length, I selected the following five PLoS ONE articles and found them to contain 18,775, 7,906, 13,424, 11,101, and 10,995 words, respectively.

   - Bocci M, Sangiuliano SJ, Sarretta A, Ansong JO, Buchanan B, Kafas A, et al. (2019) Multi-use of the sea: A wide array of opportunities from site-specific cases across Europe. PLoS ONE 14(4): e0215010.
   - Campbell BW, Marrs FW, Böhmelt T, Fosdick BK, Cranmer SJ (2019) Latent influence networks in global environmental politics. PLoS ONE 14(3): e0213284.
   - Ling T, He Y (2020) The remanufacturing evaluation for feasibility and comprehensive benefit of retired grinding machine. PLoS ONE 15(6): e0234603.

- Olsen E, Fay G, Gaichas S, Gamble R, Lucey S, Link JS. (2016) Ecosystem model skill assessment. Yes we can! PLoS ONE, 11(1): e0146467.
- You C, Lin DKJ, Young SS (2018) Time series smoother for effect detection. PLoS ONE 13(4): e0195360.

I now believe my manuscript is as long as a typical PLoS ONE article. I would be happy, however, to shorten it further if requested.

3. **Either the foundational work in Sections 1 and 2 could have been reduced to more tightly focus the paper, or the paper should have been split into two works. If the goal of the work is to provide (and evaluate & justify) a new tool for ecological management, then perhaps it would be better to split the material into (1) a paper that justifies its validity and shows its promise and (2) a suite of technical documents hosted with the open-source software. This would go some way toward dealing with the original reviewer's comments #1 and #11.**

   I have chosen to reduce the foundational content of Sections 1 and 2 so as to create a shorter, single work that offers practical guidance for those wishing to use statistical methods to perform model-based ecosystem management. I believe enough foundational material remains so that a reader can see the paper's statistical and computational approach to ecosystem management falls within the paradigm of experimental science.

4. **Re Comments 6/7: The original reviewer's point remains and could be expanded as "Perhaps the search query was too narrow and missed relevant literature". The responses only address the offered example, not the thoroughness of the original search. Re original reviewer comment #12, they have a point. There are now many approaches to achieving parallelism. What they left out was a listing of examples that _do_ support easy deployment of applications across heterogeneous devices, like Docker, which are ignored in in this paper.**

   I now report that at least three feasible alternatives to JavaSpaces exist, see lines 516-528. There, I note the trade-offs presented by Docker and make my review practical by identifying specific language commands that would be used to develop programs similar in function to the one that I exercise in the case study of Section 3.
