# Supplementary material for: Developing political-ecological theory: The need for many-task computing
Source: PLoS One. 2020 Nov 24;15(11):e0226861. doi: 10.1371/journal.pone.0226861 (PMC7685461; doi:10.1371/journal.pone.0226861)
Supplement: Attachment — Submitted filename: r3rspns.pdf [file pone.0226861.s005.pdf]

Response to Reviewers

Academic Editor Comments:

1. **The restructuring to separate the review of related work into a separate section is recommended. This will allow a clearer exposition of the objectives of the work in the introductory section.**

    I have moved all related work to Section 2, and have shortened both Sections 1 and 2. See lines 23-182.

2. **Both reviewers have recommended revision of the presentation of the algorithm in Section 2.5.2 to make it more concise and easy to follow, with any extensive commentary removed to supporting text.**

    I have considerably shortened the algorithm's text. Now, each step is one sentence. Explanatory material has been moved to a sub-section below the algorithm. See lines 395-418.

3. **The issue remains regarding the consideration given to alternative technologies. Please present the reasons for choosing JavaSpaces clearly and concisely, with relevant comparison with other methods. This has not been achieved with the brief comments already included.**

    I have rewritten and enlarged Section 3.7. I now give a neutral overview of five technologies that could be used to implement my methods. I make it clear that any of these technologies could be used to implement the article's five simulator jobs. I then compare JavaSpaces with these other four technologies and give reasons for why I chose JavaSpaces over them. See lines 460-552.

4. **Carefully consider what basic information is really required for a reader interested in this topic.**

    I have removed almost all remedial content on computing from Section 3.7, see lines 461-481.

5. **While you have made commendable efforts to make the manuscript more focussed and concise, the response to the reviewers on the length of the**

manuscript is unpersuasive.There may be papers of a similar length already published in PLOS ONE, but there are equally many that are shorter, concise and clear. It is these last two characteristics that can significantly enhance a paper.

I have removed all ancillary material and unnecessary words. I have replaced almost all quotations with paraphrasing. I have rewritten many sentences to make them shorter and more to the point. As a result, the manuscript is now 11,673 words – 10% shorter than my previous revision.

Reviewer #3 Comments:

The manuscript is certainly a large improvement on what was originally submitted. Just a few things to address:

1. **My concern is the emphasis on a particular technology, JavaSpaces. It implies that the contribution of the paper is dependant on this technology. There is no harm saying that JavaSpaces was used as the implementation of the ideas. The discussion of the computational model needs to be cast in more general terms. As such, the current Section 2.7 needs to be recast and rewritten. Also, the very basic discussions of runtime systems and compiling etc does not need to be included.**

   I have rewritten and enlarged Section 3.7. I now give a neutral overview of five technologies that could be used to implement my methods. I make it clear that any of these technologies could be used to implement the article's five simulator jobs. I then compare JavaSpaces with these other four technologies and give reasons for why I chose JavaSpaces over them.

   I have also removed almost all remedial material on computing from the section. See lines 460-552.

2. **Section 1 is far too big. The author needs to put the literature review components into a new section (Section 2) and reorganise the remaining components into Section 1.**

   I have moved all related work to Section 2, and have shortened both Sections 1 and 2. See lines 23-182.

3. **Remove paragraphs from the Abstract. It is also too long and needs to be compacted.**

   I have removed the paragraph form of the Abstract. I have also shortened it. See lines 8-19.

4. **Section 2.5.2 is not an algorithm. An algorithm is a far more precise set of simple steps that is readily translatable into code. The author needs to a) have a textual description under 2.5.2 explaining what is trying to be achieved, and then say the set of high level steps follow. I would encourage the author to try to cast this is an algorithm (there are plenty of papers out there with good examples).**

I have considerably shortened the algorithm's text. Now, each step is one sentence. Explanatory material has been moved to a sub-section below the algorithm. See lines 395-418.

Reviewer #4 Comments:

Thank you for the changes to the work. Thank you also for using latexdiff to mark up the changes. It has been the task of re-reviewing the work much easier.

1. **Regarding the length, the criticism from me and other reviewers has been that the work was overly long _given the message intended to be delivered_, and that the space devoted to background material hindered delivery of the message. The cuts to Sections 1 and 2 are welcome, and do help focus the paper better, although I strongly suspect it could be further targeted: 13000 words is a _long_ paper, and with no description of how you 'selected' those 5 other PLOS ONE papers, nor of why you consider them to be 'typical' (common, near average, near median?) length for the journal, it's unclear how this justifies the current length.**

   I agree that the issue of length is more about how many words are really needed to convey the manuscript's contributions in a concise manner rather than needing to stay under an absolute maximum length threshold. I believe that now, the manuscript delivers its content in an organized and succinct manner with all peripheral content removed. As a result of this tightening, it is now 11,673 words, 10% shorter than the previous version.

   My convenience sample of five PLOS ONE articles that I included in my previous Responses to Reviewers is nonrandom and too small to support any formal, statistical definition of a "typical" PLOS ONE article length. I used the word "typical" in an informal way therein.

2. **The core issue from previous revisions remains: is this paper expounding improved statistical modelling and evaluation techniques for this domain, or proposing a computing approach to make them tractable? Trying to do both makes the work very long, and lessens the impact of both parts.**

   I have made it more clear in the revision that for the large, agent-based models I am advocating, one cannot separate statistical assessment from statistical computing, see lines 118-131, 710-718, and 734-735. In general, I have improved my presentation so that a reader can see that these two issues are inseparable.

3. **Picking up from another review's comment and the response to it, the algorithm in 2.5.2 is still overly long and, despite the claims to have been made shorter, is actually only one sentence shorter, with several steps consisting of multiple paragraphs. That is not the typical way an algorithm is presented. Steps should be concise and clear, not laden with explanatory text. Consider splitting into an algorithm where, ideally, each step fits on 1-2 lines, with an explanation of the major tasks placed in normal paragraphs nearby.**

   I have considerably shortened the algorithm's text. Now, each step is one sentence. Explanatory material has been moved to a sub-section below the algorithm. See lines 395-418.

4. **While it's good that some text has been added to discuss alternatives to JavaSpaces, and the response claims that these alternatives are presented in a way that shows they could be used to implement the approach defined in Section 2, that is unfortunately not how the section reads. Its placement, in the middle of the discussion of JavaSpaces, is odd, since the decision of which technology to use has clearly already been made. It offers little comparison between the chosen technology (remember, we're _not_ asking you to change that) and the alternatives, and is dismissive of Docker, incorrectly tying it to MPI (it actually takes extra effort to make Docker work with MPI; it is not its most natural use). I suggest either: moving the discussion of alternatives, including JavaSpaces, to the start of the section; or adding additional critique and comparison that either (1) indicates which of these alternatives could be used with neither benefit nor loss (i.e., JavaSpaces is one, equally valid implementation choice) or (2) demonstrates why JavaSpaces is a superior choice.**

   I have removed the material concerning the use of Docker with the MPI API.

   I have rewritten and enlarged Section 3.7. I now give a neutral overview of five technologies that could be used to implement my methods. I make it clear that any of these technologies could be used to implement the article's five simulator jobs. I then compare JavaSpaces with these other four technologies and give reasons for why I chose JavaSpaces over them. See lines 460-552.

5. **Relatedly, in 3.1, could an estimate of the wallclock time if parallel com-**

puting had not been used be given, as well as an indication of the relative scale of the case study compared to other envisaged uses? This would go a long way to justifying 'the need for many-task computing', because as presented it looks like it would take 45 hours to evaluate a model that would inform planning on a 5-10 year horizon, which is actually not that long to wait for an answer.

> I have added an estimate of a sequential solution's wallclock time in order to drive home the need for MTC, see lines 710-718. Thank you for the suggestion.

6. **In 3.2, what do the parameters in Table 3 represent? It's unclear how the reader is meant to derive much meaning from numbers 168, 165, 171, 174, 183.**

> I now define each parameter in Table 3. See lines 634-643.

7. **The 'additional computational evidence [at] lines 805-811' consists entirely of references to the author's own prior work. Could any other _new_ computational evidence beyond the single case study in this paper be included?**

> I now report new computational experience. See lines 565-567. Specifically, I report on a benchmark computation I performed in September 2020 on the ability of my MDAS algorithm to find the global minimum of the classic Bukin F4 optimization test function.

8. **The moved section 4.1 seems remarkably parochial. Australian and New Zealand (presumably European, too) governments have extensive HPC facilities that are free for researcher use and hence, governmental use. Even amongst the commercial HPC providers, products like Microsoft Azure and Amazon EC2 have been completely ignored, yet they have data centres in many locations around the world. I don't object to the inclusion of the section, but if it's to be included at all it should have some more general advice.**

> I have removed the section on the funding of computer time.
